# Selenium Supplementation of Amaranth Sprouts Influences Betacyanin Content and Improves Anti-Inflammatory Properties via NFκB in Murine RAW 264.7 Macrophages

**DOI:** 10.1007/s12011-015-0429-x

**Published:** 2015-07-12

**Authors:** Malgorzata Tyszka-Czochara, Pawel Pasko, Pawel Zagrodzki, Ewelina Gajdzik, Renata Wietecha-Posluszny, Shela Gorinstein

**Affiliations:** Department of Radioligands, Medical College, Jagiellonian University, Krakow, Poland; Department of Food Chemistry and Nutrition, Medical College, Jagiellonian University, Medyczna 9, 30-688 Krakow, Poland; Faculty of Health and Medical Science, Andrzej Frycz Modrzewski Krakow University, Krakow, Poland; Department of Nuclear Physical Chemistry, Institute of Nuclear Physics, Krakow, Poland; Department of Analytical Chemistry, Faculty of Chemistry, Jagiellonian University, Krakow, Poland; The Institute for Drug Research, School of Pharmacy, The Hebrew University, Hadassah Medical School, Jerusalem, Israel

**Keywords:** Selenium-enriched amaranth sprouts, Betacyanin, Murine macrophage cell line (RAW 264.7), NFκB translocation, Interleukin 6

## Abstract

Sprouts contain potent compounds which while influencing crucial transduction pathways in cell reveal anti-inflammatory and anticancer activities. In this study, we report the biological activity for seeds and colourful sprouts of four types of edible amaranth, as amaranth has recently attracted interest due to its appreciable nutritional value. MTT assay conducted for the amaranth seeds and sprouts did not show any adverse effect on the viability of murine RAW 264.7 cells. As amaranth accumulates selenium, the sprouts were supplemented with this trace element (10 mg/L; 15 mg/L Se as sodium selenite) while growing. Selenium concentration in sprouts was observed to be significantly correlated with betacyanins content of the tested species. The amounts of Se and betacyanins in sprouts varied for various *Amaranth* species. In the present study, *Amaranthus cruentus* sprouts with the highest betacyanins (19.30 ± 0.57–28.85 ± 2.23 mg of amaranthin/100 g of fresh weight) and high total selenium (22.51 ± 1.57–1044.75 ± 73.08 μg/L in methanol extracts) content prevented NFκB translocation to the cell nucleus and subsequently exerted an anti-inflammatory effect by significant decreasing inflammatory interleukin 6 production (587.3 ± 34.2–710.0 ± 88.1 pg/mL) in the cell culture of activated RAW 264.7 macrophages (vs LPS control 1520 ± 114 pg/mL).

**Electronic supplementary material:**

The online version of this article (doi:10.1007/s12011-015-0429-x) contains supplementary material, which is available to authorized users.

## Introduction

Daily nutrition may provide a variety of bioactive nutrients that may demonstrate physiological benefits and reduce the risk of chronic diseases. The new direction in human nutrition involves discovering and developing a new kind of food, so called functional food, whose functions exceed by far the basic nutritional ones. One of the prospective beneficial effects of the functional food on the organism is that it exerts a positive influence on inflammatory processes. It has been proven that vegetables, fruits and grains may present anti-inflammatory activity and their effect is associated mostly with the content of phytochemicals such as phenolic acids, flavonoids or anthocyanins [1, 2]. Since chronic inflammation is involved in aetiology of several malfunctions such as cardiovascular diseases, neurodegenerative disorders, diabetes mellitus and cancer, the investigation is highly required. Sprouts, the ‘new kind’ vegetables, which contain potent antioxidant compounds which act as free radical scavengers and, influencing crucial transduction pathways in cell, have been reported to reveal anti-inflammatory and anticancer activities [3–5].

Amaranth, a colourful pseudocereal, has recently attracted live interest due to its appreciable nutritional value. Several species of amaranth have edible seeds, sprouts and leaves. They are rich in unsaturated fatty acids, squalene and minerals, have higher protein content than most cereals do and are essentially gluten free. Thus, the plant family has become an important crop for agronomy and food industry. Especially, amaranth sprouts have recently received attention as a new product that might fortify human diet with beneficial components such as natural antioxidants, and it may impose beneficial effects on several aspects of human health [6]. Several studies reported appreciable bioactive properties of amaranth seeds [7, 8]; however, little is known about the biological activity of amaranth sprouts.

As the intensive colour of amaranth results from quite considerable betacyanins concentration in plant, it can provide an alternative phytochemicals source to beetroot. Betacyanins express red-violet colour, and so with regard to their chemical structure, they belong to betalains, i.e. water soluble pigments containing nitrogen. Natural products derived from *Amaranthaceae* family contain pigments such as amaranthin, isoamaranthin, iresinin, isoiresinin I, celosianin I, isocelosianin I, celosianin II and isocelosianin II [9, 10]. In *Amaranthus cruentus*, only amaranthin and isoamaranthin were identified as representatives of amaranth-type betacyanins. Betanin and isobetanin as betanin-type betacyanins were found in *Amaranthus powellii* [9]. This group of natural pigments characteristic for *Amaranthaceae* family have been under close scrutiny due to their significant antioxidant activities. Amaranth betacyanins were also reported to act against oxidative stress-related disorders, though the mechanisms of their action in organism are not well documented [9–11]. Gallic acid, p-hydroxybenzoic acid, vanillic acid and rutin were found to be the most critical compounds for amaranth antioxidant properties in seeds and sprouts, out of all polyphenols though data on betacyanins are poor. The antioxidant activity of amaranth products, such as seeds, sprouts, cereals, flakes or bread, was confirmed with in vitro evaluation [12, 13] in vivo experiments, by using the rat model [14, 15].

On the other hand, the new trends in food production and consumption promote selenium rich plant products [16, 17]. Amaranth sprouts were reported likely to accumulate selenium during their growth. Still, selenium is toxic to many plants valuable for human nutrition. The number of plants capable of accumulating selenium compounds applicable as a component of daily diet is very limited and includes *Oonopsis sp.*, *Xylorhiza sp.*, *Neptunia sp.*, *Astragalus sp.*, *Morinda sp.* and *Stanleya sp*. Nevertheless, one of these species meets dietary requirements, as for none safe consumption is guaranteed [18]. Hence, hope for *amaranth* sprouts to fill the gap arise. Among commonly used selenium compounds, selenite is found to be four times more toxic than selenomethionine, while for the latter one LD_50_ depends on the administration route and is equal to several mg/kg body mass. Higher doses of selenium compounds administered to animals cause cardio-respiratory effects to occur, hind limb paralysis, loss of body hair and even death [19]. Symptoms for selenium intoxication in humans include brittle hair and nails with apparent surface changes, pruritic rashes, garlic breath, nausea, dental caries, skin lesion and neurological impairment [19–21]. Epidemiologic data from China suggest that daily intake of 750–850 μg sets the upper limit of safe dose [20].

Selenium revealed anti-inflammatory and anticancer activity in in vitro as well as in vivo studies [22]. It was indicated that selenium derivatives modulate various signal transduction pathways influencing cell signalling [22] and regulation of cell NFκB pathway [23]. The transcription factor NFκB plays a crucial role in the response of cell to the inflammatory signalling. In macrophages activated by an external or internal stimuli (IL-1, LPS, TNF-α, ROS), the rapid activation of NFκB involves its translocation into nucleus, where it may regulate the transcription of pro-survival factors and immunity effectors such as IL-6 [24, 25]. The influence mechanism on immune system for food components includes the influence on cytokines and inflammatory mediators (PGE_2_, NO) [2]. NFκB as a master regulator of complex cell functions provides a very attractive therapeutic target for anti-inflammatory pharmacological and nutritional interventions.

The study was primarily aimed at (1) determining the biological activity for seeds and sprouts of several amaranth species in relation to their betacyanins concentration and (2) checking their potency to modulate the immunological response. The sprouts were additionally supplemented with selenium. The biological activity for seeds and sprouts extracts of several amaranth species, namely *Amaranthus cruentus*, *Amaranthus caudatus*, *Amaranthus paniculatus* and *Amaranthus tricolor* are reported. The effect of amaranth seeds and sprouts on the viability of murine RAW 264.7 cells was measured with MTT assay. To assess the Se influence on amaranth sprouts properties, the sprouts were supplemented with Se at doses of 10 and 15 mg/L. After harvesting, Se and betacyanins levels in plant material supplemented with Se while growing and without Se supplementation were measured. The potency of seeds and sprouts extracts of *A. cruentus* to modulate the immunological response after TNFα activation of RAW 264.7 macrophages was evaluated through NFκB translocation. The effect of incubation with *A. cruentus* extracts was confirmed by measuring the concentration changes of IL-6 released by RAW 264.7 cells stimulated with LPS.

## Material and Methods

### Material

Four species of edible amaranth seeds were used in sprouting. Voucher specimens were deposited in the Department of Food Chemistry and Nutrition, Faculty of Pharmacy, Medical College, Jagiellonian University with reference number AMCRU/PP/PL 1039 to *A. cruentus* (harvested in Poland), AMCAU/PP/PL 1040 to *A. caudatus* (harvested in Netherlands), AMPAN/PP/PL 1041 to *A. paniculatus* (harvested in Poland), AMTRI/PP/PL 1042 to *A. tricolor* (harvested in China). The four kinds of amaranth seeds were selected for sprouting as they are edible, widely available on the market and popular in cuisine over the world to replace flour, grains and vegetables [13, 26, 27]. Some of amaranth species, in particular, *Amaranthus retroflexus* and *Amaranthus hybridus*, may be harmful for living organism due to high levels of oxalates and nitrate/nitrite that may cause acute renal necrosis and perirenal oedema in ruminants, swine and horses [28].

### Sprouts Harvesting

The amaranth seeds were immersed in tap water (HCO_3_^−^ 131.06 mg/L; F^−^ 0.07 mg/L; Mg^2+^ 5.62 mg/L; Ca^2+^ 41.69 mg/L; Na^+^ 9.65 mg/L) and tap water with two selenium concentration levels (10 mg/L; 15 mg/L Se as sodium selenite (Fluka)) for 3 h and then placed in clay vessels. As selenite does not affect agronomic species germination up to the concentration of about 30 mg Se/L [29], we decide to establish the selenium doses at half and one third of such a threshold value. Sprouts were grown for 6 days after seeding at the controlled temperature of 24 ± 2 °C and watered daily with tap water, and selenium supplemented water. All cultures were stored in natural light conditions. Selenium tolerance for various edible amaranth seeds was screened to evaluate the most appropriate amaranth genus for selenium enrichment [6]. The applied harvesting procedure for amaranth sprouts is described elsewhere [6].

### Extracts Preparation and Analysis of Betacyanin

Seeds and sprouts samples of 0.2 g of fresh weight were homogenized in a cold mortar with 1.5 mL of cold distilled water. The procedures associated with betacyanin extraction were performed at 2–4 °C according to Cao et al. [30]. The obtained homogenates were transferred into tubes and centrifuged at 18.000*g* at 4 °C for 15 min. Then, 0.6 mL of supernatant was shaken with 1.0 mL of chloroform (Sigma-Aldrich) for 15 min and centrifuged at 4 °C for 30 min. The aqueous phase absorbance was measured with spectrophotometer (Jasco V-530, Japan) at 536 nm [31]. Betacyanin content in amaranth seeds and sprouts was expressed as mg of amaranthin/100 g fresh weight. Each sample was evaluated in three replicates, and the results were expressed as mean value ± SD.

### Extracts Preparation for Evaluation of Selenium Concentration and for Cells Study

Samples of four amaranth seeds, namely *A. cruentus*, *A. caudatus*, *A. paniculatus*, *A. tricolor* and fresh sprouts of the species (4 g), selenium free and supplemented with selenium at two doses of 10 and 15 mg/L, were extracted with 35 mL of hot methanol (Dor-Chem) for 3 h. The obtained extracts were decanted, centrifuged and stored in darkness in a freezer at −20 °C. For MTT analysis, and determination of NFκB nuclear translocation and IL-6 expression in RAW 264.7, cells dry methanol extracts in concentration of 10 mg/mL were obtained under reduced pressure and subsequently were dissolved in DMSO (Sigma-Aldrich).

### Selenium Concentration

The 12-positional microwave system MARS X (CEM, Matthews, USA), equipped with temperature (RTP-300) and pressure (ESP-1500 Plus) sensors, was used for sample preparation. The system allowed programmable control of pressure and temperature. The sample portion of 0.3 mL (methanol extract) in 7 mL of concentrated HNO_3_ was digested at the maximum temperature of 200 °C. The digestion conditions applied for the microwave system were 16 min/960 W and 8 min/1080 W. Following digestion, the sample solution was cooled in the air to 25 °C and purged under nitrogen flow for 10 min. The Mini-vap device (Sigma Aldrich, Germany) was used to remove gaseous products with nitrogen. Finally, the sample was transferred into a 25 mL volumetric flask, mixed with 12.5 mL of 6 mol/L HCl and diluted to the mark with deionized water. For selenium concentration analysis, a double-channel atomic fluorescence spectrometer AFS-230 (Beijing Haiguang Instrument Co., China) with flow hydride-generation system was used. A cathode lamp (Se-HCL) designed for AFS measurements, operating at pulsed current of 100 mA, was used as the light source. The argon-shielded gas flow and the carrier gas flow were 800 and 500 mL/min, respectively. The atomization process occurred in the Ar-H_2_ flame at 200 °C. The signals corresponding to selenium content in plant extracts were recorded and processed in the peak area mode with the use of an IBM 586 computer (Beijing Haiguang Instrument Co., China).

### Cell Culture

RAW 264.7 macrophage cell line (TIB 71, adherent cells from *Mus musculus*) from American Type Cell Culture collection, ATCC, USA, were cultured in Dulbecco’s Modified Eagle’s Medium (DMEM, ATCC, USA) supplemented with 10 % *v*/*v* FBS, 100 IU/mL penicillin and 0.1 mg/mL streptomycin (Gibco Laboratories, NY, USA). The cells were kept at 37 °C in a humidified atmosphere of 5 % CO_2_—95 % air under standard conditions. The number of cells was assessed by automatic cell counter Countess (Gibco Laboratories, NY, USA). The cell culture morphology was investigated by means of inverted light microscope (Olympus CKX 41SF-5 microscope, Olympus, Germany).

### Cell Viability Assay

The viability of RAW 264.7 cells incubated with variable concentrations of amaranth extracts was assayed with MTT test. Cells (5 × 10^5^ cells/well) were seeded onto the 96 multi-well plates (Sarstedt, Numbrecht, Germany) and kept for 24 h as adherent cultures at 37 °C. Then, different dry methanol amaranth extracts dissolved in DMSO were added to the appropriate cell wells, and incubation was performed for 24 h. Six concentrations of amaranth extracts: 1 mg/mL; 500 μg/mL; 100 μg/mL; 10 μg/mL; 1 μg/mL; 0.1 μg/mL of medium were tested. The cells cultured in medium were used as positive control (100 % of growth), whereas the cells incubated with addition of 20 mM/L hydrogen peroxide to medium provided the negative control (0 % of growth). The test was conducted as described previously [32]. The absorbance was measured at 550 nm (the reference wavelength of 690 nm) using Universal Microplate Reader ELX800NB (Bio-Tek Instruments INC, Winooski, VT, USA). The following formula was applied to report the results: (average OD value over three measurements for each experimental group/average OD value of control group) × 100 %. Each experiment was repeated in triplets.

### Translocation of Transcription Factor NFκB

RAW 264.7 cells were seeded onto plates at the density of 2 × 10^6^ cells/well and kept overnight. Then, the cells were incubated for 24 h with 10 μg/mL of *A. cruentus* extracts as follows: (a) seeds, (b) Se free sprouts, (c) Se supplemented sprouts (10 mg Se/L added to water) and (d) Se supplemented sprouts (15 mg Se/L added to water). The cells with addition of vehicle to the media served as the controls. The same experiment was performed for all the experimental groups exposed overnight to extracts as described above, but following 24 h incubation 10 ng/mL of TNFα was added to each of the experimental group in order to stimulate NFκB translocation from cytoplasm to the nucleus. Then, the incubation was continued for the next 24 h.

The cells of all the experimental groups were lysed for immunoblot analysis in buffers (Thermo Fisher Scientific Inc., USA) with a protease inhibitor cocktail (Merck, Darmstadt, Germany). Nuclear and cytoplasm fractions were separated from cells with organelles extraction kit (Merck, Darmstadt, Germany). The protein samples were resolved via standard SDS-PAGE and then transferred to PVDF membranes. The membranes were blocked with buffer contained 1 % casein in TBST (20 mM/L of Tris-hydrochloride, pH 7.5, 150 mM/L NaCl, 0.05 % Tween 20). Immunoblotting was performed using a 1:500 dilution of an anti-NFκB antibody (Cell Signalling, Danvers, MA, USA), a 1:250 dilution of an anti-phospho NFκB (Cell Signalling Technology, USA), followed by a 1:6000 dilution of an HRP-linked anti-mouse secondary antibody (Santa Cruz Biotech., Santa Cruz, CA, USA). GAPDH (1:1000, Cell Signalling, Danvers, MA, USA) and histone H4 (1:250, Cell Signalling, Danvers, MA, USA) were the loading controls for cytoplasmic and nuclear fractions, respectively. The specific immunoreactivity was demonstrated by enhanced chemiluminescence. The immunoblots were developed using the Super Signal West Pico Chemiluminescent Substrate Kit (Pierce Chemical, Rockford, IL, USA) using Gel Logic Imaging System 1500 (Kodak; Molecular imaging System Corestea Health Inc., Rochester, NY, USA). Total protein was measured by the Bradford method using bovine serum albumin as a standard. Each experiment was repeated three times.

### Interleukin 6 (IL6) Release

RAW 264.7 cells were seeded onto 96 multi-well plates (5 × 10^5^ cells/well) and exposed to an amaranth extracts for 24 h as described above. The cells with addition of vehicle to the media served as the controls. Following incubation with extracts, 10 ng/mL of LPS was added to every culture well to increase NFκB activity and IL-6 release from the activated macrophages and the incubation was continued for the next 24 h. IL-6 release (pg/mL) was determined in the culture medium with an ELISA quantitative test (Gen-Probe Inc., USA). The assay was performed according to the manufacturer protocol with a universal microplate reader ELX800 MB. The absorbance was measured at 450 nm; the reference was 630 nm. Each experiment was repeated three times.

### Statistical Analysis

The experimental data are shown as means ± SD. Analysis was performed with one-way ANOVA followed by the Duncan post hoc test and the significant difference was set at *p < 0.05, ** *p* < 0.01.

The multivariate principal component analysis (PCA) method was applied to address the issue of interactions between the investigated parameters. It was assumed that the parameters with absolute values of their weights, i.e. coordinates, higher than 0.3 with respect to the first or second principal component were considered to be correlated [33]. To gain quantitative evidence weight of the results, for pairs of the considered parameters algebraic products of their corresponding weights and the corresponding angle cosine, i.e. the angle determined by two lines on the PCA plot connecting the origin of the coordinative system with the coordinates of both parameters, were calculated. The cosine multiplication is a correction factor for relative position of these parameters in a multivariate space—for orthogonal states, no correlation occurs, whatever big values their weights take. The results of such ‘corrective’ calculations are called correlation weights (CW).

Cluster analysis (CA) was chosen to group objects, i.e. our samples, into similar categories. This analysis was performed after data standardization. Ward agglomeration procedure and the Euclidean distance were assumed for the grouping method and the distance function, respectively. The number of distinctive clusters was calculated with the Mojena’s rate applied as criterion.

Statistical calculations were carried out using the commercially available packages SPSS Statistics 22 (IBM, USA) and Statistica PL v.10 (StatSoft, USA) and the software delivered by MP System Co. (Poland).

## Results and Discussion

### Betacyanins Concentration in Amaranth Seeds and Sprouts

*Amaranthaceae* family has recently attracted interest due to its appreciable nutritional value and considerable concentration of phytochemicals combined with a high biological activity of various amaranth products. The precise biological action of several compounds abundant in amaranth, such as betacyanins, is poorly understood. In the present study, the concentrations of betacyanins in seeds and sprouts of edible amaranth species, namely *A. cruentus*, *A. caudatus*, *A. paniculatus* and *A. tricolor* were measured. The total concentrations of betacyanins recalculated for the amount of amaranthin per 100 g of FW are shown in Table 1. The seeds contained significantly (*p* < 0.01) lower amount of betacyanins when compared to sprouts. The betacyanins concentrations in the amaranth seeds ranged from 1.09 ± 0.06 mg to 6.00 ± 0.28 mg (expressed as mg amaranthin/100 g FW). The highest amount of the pigments was presented in seeds of *A. caudatus*, compared to the remaining groups. The differences between the others seed samples were not statistically significant. In the control sprouts, grown without Se supplementation, the highest amount of betacyanins was present in *A. cruentus* material (28.85 ± 2.23 mg/100 g FW), whereas the lowest one in *A. tricolor* (9.23 ± 0.13 mg/100 g FW, the difference was statistically significant at *p* < 0.05). All the tested sprouts samples of *A. caudatus* and *A. paniculatus* contained similar amount of betacyanins (about 14 mg/100 g FW).

**Table 1:** The content of betacyanins in amaranth seeds and sprouts, expressed as mg of amaranthin per 100 g of fresh weight (FW) (*n* = 3)

Sample	Seeds	Sprouts (Aq)	Sprouts (10 mg Se/L)	Sprouts (15 mg Se/L)
*A. cruentus*	1.09 ± 0.06	28.85 ± 2.23	21.92 ± 0.92*	19.30 ± 0.57*
*A. caudatus*	6.00 ± 0.28	14.75 ± 0.74	14.30 ± 0.16	13.53 ± 0.27
*A. paniculatus*	1.15 ± 0.19	14.76 ± 0.75	14.31 ± 0.20	13.50 ± 0.30
*A. tricolor*	1.41 ± 0.05	9.23 ± 0.13	6.95 ± 0.05*	7.20 ± 0.21*

*p < 0.05 (significant difference compared with control (sprouts watered only with water))

As reported by Cai et al. [9], the analysis of betacyanins content is usually conducted in fresh plant material including amaranth sprouts [31], while the analysis of food with addition of *Amaranthus* pigment, for jelly and ice cream that contained dried *A. cruentus*, genotype Cr072, was also reported [31]. It was demonstrated that total betacyanins in the various wild amaranth species may range from 46 to 199 mg/100 g FW, while cultivated amaranth species contained significantly more betacyanins than the wild ones [9]. The influence of physical variables such as light, as well as chemical factors such as methyl jasmonate, ethylene or salicylic acid, on betacyanins content in *Amaranthus mangostanus* seedlings was also studied by Cao et al. [30]. In particular, betacyanins synthesis was concluded to be enhanced by light and methyl jasmonate treatment.

### Accumulation of Se in Amaranth Seeds and Sprouts

Under this study, amaranth sprouts were demonstrated to be a likely source of betacyanins and Se, when supplemented with selenium ions while growing. Amaranth sprouts capability to accumulate Se, reported before [6], was found to be very encouraging with regards to using amaranth sprouts as functional food. The amaranth sprouts for *A. tricolor* watered with addition of 15 mg/L of Se (data not shown here) were measured to accumulate 82 ± 1.8 mg Se/kg dry weight [6]. Under present experimental conditions, the sprouts of *A. caudatus* and *A. cruentus* were found to accumulate much higher amounts of this trace element, namely 1044.8 ± 73.1 μg Se/L and 1200.9 ± 150.1 μg Se/L of methanol extracts, respectively. The amounts of Se in samples of all the tested amaranth species watered with water (0 mg Se/L) were significantly lower than in samples with addition of selenium (see Table 2). Two sprouts species (*A. paniculatus*, *A. tricolor*) supplemented with 15 mg Se/L indicated a higher accumulation efficiency than the ones supplemented with 10 mg Se/L, as the observed increase of selenium concentrations in their stems was much higher than resulting solelt from its increased concentration in the growing media. The lowest concentrations of Se were observed in seeds of the tested species. Lintschinger et al. [34] evaluated selenium influence on germination for eight edible seeds of alfalfa, barley, millet, oat, pea, rye, sunflower and wheat. Unfortunately, barley, millet, oat, pea and rye sprouts showed a major reduction in the germination rates with selenium doses present. Sprouts of wheat and alfalfa occurred to be less resistant, and selenium content was enriched up to the concentrations of 100 and 150 mg of Se/kg of dry mass, with the best results obtained for sunflower sprouts where selenium concentrations up to 900 mg of Se/kg of dry mass were recorded. Mung bean sprouts (Vigna radiate) whose practical application (it contained 223.45 mg Se/kg) was evaluated by Chinrasri et al. [35], provide another good selenium source. Also, Sugihara et al. [36] cultivated 28 sprouts species from 10 families in a high selenium environment. The highest selenium concentration of over 30 μg/g wet weight was obtained for Chinese cabbage, turnip, nozawana (a type of turnip), broccoli, kintoki (a Japanese carrot) and parsley.

**Table 2:** The total selenium concentration (μg/L) in methanol extracts (*n* = 3) of edible amaranth seeds and sprouts samples (*Amaranthus cruentus*; *Amaranthus caudatus*; *Amaranthus paniculatus*; *Amaranthus tricolor*) after 6 days of growing in water based solutions (0, 10 and 15 mg Se/L)

Sample	Seeds	Sprouts (Aq)	Sprouts (10 mg Se/L)	Sprouts (15 mg Se/L)
*A. cruentus*	14.21 ± 0.99	22.51 ± 1.57	842.58 ± 58.98	1044.75 ± 73.08
*A. caudatus*	5.00 ± 0.35	15.67 ± 1.10	840.16 ± 75.20	1200.92 ± 150.13
*A. paniculatus*	8.55 ± 1.70	36.5 ± 2.39	329.58 ± 23.10	920.75 ± 69.85
*A. tricolor*	4.50 ± 0.75	29.42 ± 2.24	723.83 ± 19.89	1256.33 ± 168.90

### The Effect of Se Supplementation on Betacyanins Concentration in Amaranth Seeds and Sprouts

Since Se plays an essential role in plants development, it was much of an interest to check whether this trace element may influence betacyanins concentration in amaranth species. The Se influence on betacyanin level in *A. cruentus* and *A. tricolor* seeds and sprouts was clearly proven in our study (Table 1). Selenium caused significant reduction in betacyanin level for both species, while for the other two, no changes were observed. Whether the mechanism of such interaction is intrinsic to specific sprout species remains to be investigated.

Se impact on betacyanin production in plants is to the best of our knowledge reported for the first time. As the role of Se in synthesising such phytochemicals and in metabolism is poorly understood, more detailed studies to explain their mechanisms are required.

### Effect of Amaranth Seeds and Sprouts on RAW 264.7 Macrophages Viability/Proliferation

It requires strong evidence of safety to recommend amaranth sprouts as a new nutritional product for humans. Therefore, in the present study, cytotoxicity of seeds and sprouts extracts was assessed. MTT cell viability/proliferation assay is one of the essential tests performed while evaluating the effects of sprouts on mammalian cells. Kim et al. [3] estimated antiproliferative action of mung bean sprouts on human pulmonary carcinoma cell line (Calu-6) and human gastric carcinoma cell line (SNU-601). Rutabaga sprouts examinations revealed cytotoxic activity in human hepatoma HepG2 cells culture [4]. Yoshida et al. [37] reported antiproliferative activity of Se-enriched radish sprouts against tumour cells in vitro. In the present study, RAW 264.7 murine macrophages have been used to the effect of *A. cruentus A. caudatus*, *A. paniculatus* and *A. tricolor* on cells viability. RAW 264.7 cells were consistently used in experiments on the influence of plant chemicals on cells viability and immune reactions, and specifically, this cell line retains essential immune properties characteristic for macrophages in vivo.

The results obtained with MTT assay confirmed that only 24 h incubation of cells with the highest concentration of extracts (1 mg/mL) reduced proliferation of cells. The addition of 1 mg/mL of each extract significantly decreased cells viability for all the tested amaranth seeds and all the sprouts; only 10 % of the cells were alive and biochemically active in all the cultures, see Online Resource, while extracts of the concentration of 10 μg/mL or less did not tend to decrease cells viability. Tendency for the tested amaranth seeds and sprouts to influence cells viability was similar.

*A. paniculatus* and *A. tricolor* sprouts grown in water without Se were less toxic to RAW 264.7 cells than the corresponding seeds. The Se supplemented growth of sprouts caused cells viability to decrease effectively for the dose of 500 μg/mL of extracts than for the same dose of extracts for sprouts grown without Se. The effect was particularly apparent for *A. cruentus* and *A. paniculatus*. On the other hand, the addition of 1 mg/mL *A. paniculatus* seeds extract reduced the amount of living cells to less than 10 %, while about 20 % of the cells survived in Se supplemented sprouts cultures at the same dose (see Online Resource C). The effect of minor cytotoxicity of *A. paniculatus* coincides with the lowest amount of Se accumulated in these sprouts (Table 2). The reason for such irregularities is not clear to us at the moment.

Kim and Milner [38] demonstrated that Se can retard cell proliferation and promote apoptosis in vitro. This observation is in accordance with the effect of *A. caudatus* sprouts watered with Se (Online Resource B), which accumulated the highest amount of Se, and at concentration of 500 μg/uL caused the highest decrease in cell proliferation (10 mg/mL of supplemented Se, see Online Resource B).

The data indicate that at concentration below 10 μg/mL amaranth sprouts with/without Se had no toxic effect on RAW 264.7 cells. A similar effect was also observed in the study on the influence of rutabaga sprouts on CHO-K1 cells [4].

### The Correlation Structure of Parameters

**Fig. 1:**
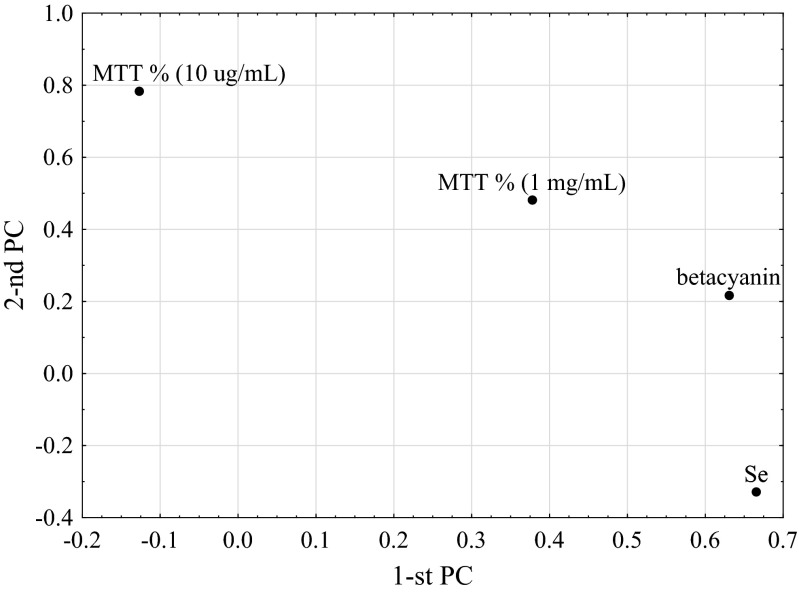
Plot of the first two principal components

The statistically significant PCA model with two significant components was derived. The eigenvalue for the first component was 1.41, and 1.12 was for the second. The first two components accounted for 32.8 and 29.0 % of the variance in the original parameters. The plot for the first two principal components is shown in Fig. 1. Se concentration was shown to be positively correlated with betacyanin concentration (CW = 0.296), and the latter parameter was also correlated with MTT% (1 mg/mL) (CW = 0.200). For two parameters, resulting from MTT assay, namely MTT% (10 μg/mL) and MTT% (1 mg/mL), a high positive correlation (CW = 0.330) was also found. The revealed positive correlation between Se and betacyanin levels can be contributed to the fact that all the samples (i.e. both seeds and sprouts) were taken into account in PCA analysis; seeds with selenium amount several times lower also contained much fewer betacyanins as compared with the sprouts, regardless of sprout Se supplementation.

### The Cluster Analysis of Samples

**Fig. 2:**
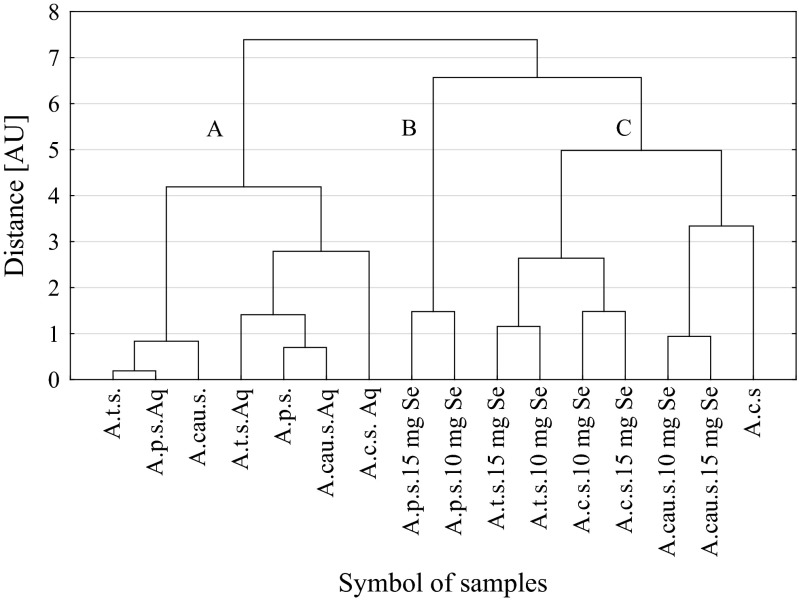
Dendrogram of similarity of samples. Mojena’s rate: *d* = 5.44. Meaning of abbreviations: *A. c.*—*A. cruentus*; *A.cau.*—*A. caudatus*; *A. p.*—*A. paniculatus*; *A. t.*—*A. tricolor*; *s.*—seeds; *s. Aq*—sprouts watered with water without Se; *s.10 mg Se*—sprouts watered with 10 mg Se/L water; *s.15 mg Se*—sprouts watered with 10 mg Se/L water

It was possible to distinguish three different clusters of samples (A, B, C) (Fig. 2) by means of the cluster analysis. Obviously, members of each cluster display some common distinctive characteristics. Cluster B was the most homogenous one and consisted of only two samples of *A. paniculatus* sprouts treated with either 10 or 15 mg Se/L. Cluster C contained all the other sprouts treated with selenium and, additionally, *A. cruentus* seeds. A specific regularity was found for this cluster as the three sub-clusters were formed by the sprouts of the same species that differed only in selenium concentration in the growing media. On the contrary, cluster A contained all the other samples that were not treated with selenium. Hence, with the only exception for *A. cruentus* seeds, the cluster structure of studied samples followed our expectations and clearly reflected different sprouts characteristics in the original multivariate space of parameters. Therefore, it is worth noting that the Wards’ minimum-variance method of clustering was successfully applied. This method tends to join clusters with a small number of samples and is biased towards finding clusters with roughly the same number of samples. The three resulting clusters remain reasonably established and, apparently, there is no random association when considering their composition.

### Effect of Amaranth Seeds and Sprouts on Translocation of Transcription Factor NFκB into the Cell Nucleus in RAW 264.7 Macrophages Stimulated with TNFα

The present study was also aimed at evaluating amaranth seeds, normal sprouts and Se supplemented sprouts capabilities to modulate immune response in cells and exert an anti-inflammatory activity. In the subsequent experiments, only *A. cruentus* seeds and sprouts were tested. *A. cruentus* samples combined the highest amount of betacyanins with the appreciable ability for Se accumulation and minor antiproliferative effect in the cultured cells when compared with the other amaranth species. Additionally, *A. cruentus* is of high nutritional value and significant importance for gluten free food production [12, 13].

Several studies reported dietary betacyanin to modulate the biochemical processes and regulate pathways essential for cell survival though the exact mechanisms remains unclear. The overall effects of betacyanins were associated with their ability to scavenge free radicals [9]. We considered that these bioactive particles may exert the effect not only via diminishing the oxidative stress but also through a direct effect on the signal transduction pathways. ROS induced by TNF-α are capable to activate nuclear factor NFκB, which results in the enhanced production of pro-inflammatory cytokines such as IL-6. The activation of NFκB by TNF-α and LPS involves a classic pathway, which causes the transportation of p65/p50 proteins to the nucleus and the subsequent changes in particular gene transcription [39, 40]. Therefore, the cellular localization of p65/p50 subunits is considered essential for regulating cell response via NFκB.

**Fig. 3:**
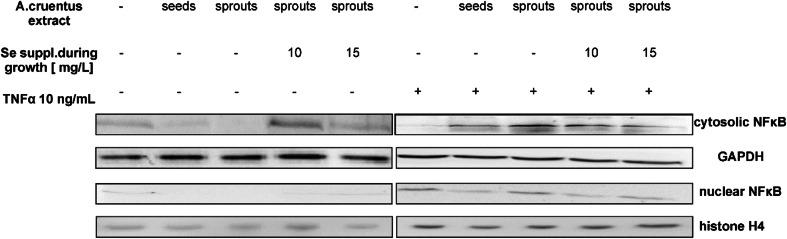
Effect of *A. cruentus* seeds and sprouts extracts in RAW 264.7 macrophages after 24 h of extracts pre-treatment (10 μg/mL). In the *right panel*, TNF-α induced translocation of NFκB, in the *left panel*, unstimulated cells. Western blot was performed to detect cytosolic (normalized to GAPDH) and nuclear (normalized to histone H4) levels of p65 subunit. Experiment was repeated three times with similar results

To investigate whether *A. cruentus* seeds and sprouts affect the nuclear translocation of NFκB, Western blot analysis of p65 subunit of NFκB was carried out in unstimulated (Fig. 3, left panel) and stimulated (Fig. 3, right panel) RAW 264.7 macrophages. As shown in the right panel of Fig. 3, the control RAW 264.7 macrophages treated with TNF-α caused dephosphorylation of protein and further translocation of p65 subunit of NFκB from cytoplasm to the cell nucleus. The amount of nuclear NFκB was markedly decreased upon pre-exposure to seeds and sprouts extracts, and the effect was more pronounced for sprouts (Fig. 3, the right panel). Since Se anti-inflammatory effect was reported [22], under this study, we combined the experiments on the antioxidant effects of amaranth sprouts supplemented with Se to find out whether such modification may result in enhancing anti-inflammatory action of amaranth. The results demonstrated that 24 h pre-treatment of RAW 264.7 macrophages with *A. cruentus* sprouts grown with or without of Se prevented the translocation of the transcription factor NFκB in cell to the same extent. Notably, Se supplementation of sprouts caused expression of cytosolic p65 subunit of NFκB in unstimulated RAW 264.7 macrophages to increase (the left panel of Fig. 3). The amount of p65 NFκB protein in cells was higher, but no influence on the process of NFκB translocation was observed.

The obtained data on inhibiting NFκB translocation by *A. cruentus* seeds and sprouts extracts suggest that amaranth has anti-inflammatory effect in stimulated mammalian macrophages. The anti-inflammatory potency of amaranth protein extract was also reported elsewhere [7].

### The Effect of Amaranth Seeds and Sprouts on IL-6 Release in RAW 264.7 Macrophages Stimulated with LPS

As discussed previously, it was well established that betacyanin acts against oxidative stress. In vivo ROS, cytokines and several immune mediators are involved in the stimulation of inflammatory response in macrophages. During the inflammation process, the internal (TNFα) and external (LPS) stimuli also trigger macrophages to release IL-6 through NFκB activation, which in turn contributes to the inflammatory reaction [41].

In our experiments, the activation of RAW 264.7 cells with 10 ng/mL LPS caused the synthesis of a pro-inflammatory cytokine, IL-6 (Table 3) to enhance. It was demonstrated that 24 h pre-treatment of RAW 264.7 macrophages with extracts prepared from *A. cruentus* seeds and sprouts (grown with and without of Se) led to a significant decrease in IL-6 concentration in culture medium, in contrast to the positive control cultures (incubated with LPS) where a substantial IL-6 release from cells was observed. IL-6 concentrations in all the cell cultures incubated with *A. cruentus* were significantly lower at *p* < 0.05 than in the medium of stimulated control cells. RAW 264.7 cells incubated with extracts of sprouts supplemented with 15 mg/L of Se while growing secreted a significantly smaller amount of IL-6 into the culture medium than for cells incubated with seeds extracts. As Western blot analysis of NFκB translocation did not resolved whether Se supplemented to growing sprouts might cause any differences in the transcription factor activation to occur, the existing difference between the action of *A. cruentus* seeds and 15 mg/L Se supplemented sprouts extracts on IL-6 release may be contributed to an unknown mechanism triggered by Se in the stimulated macrophages. Considering the mechanism of Se action in the cell where this trace element is the essential component of antioxidant enzymes, e.g. thioredoxin reductase (TR) and glutathione peroxidase (GPX), it may inhibit inflammation both by reducing ROS level and by decreasing cell lipid peroxidation [38]. Kretz-Remy et al. [42] suggested that increase of cellular GPX (cGPX) activity can interfere with NFκB activation with concomitant minor influence on gene expression or protein stability.

**Table 3:** Effect of *A. cruentus* seeds and sprouts extracts on LPS (10 ng/mL) induced IL-6 release [pg/mL] from RAW 264.7 macrophages after 24 h of extracts pre-treatment (10 μg/mL)

Sample	Positive control (LPS)	Negative control	Seeds	Sprouts (Aq)	Sprouts (10 mg Se/L)	Sprouts (15 mg Se/L)
IL-6[pg/mL]	1520 ± 114	25.3 ± 4.65	846.0 ± 73.45	710.0 ± 88.1	674.0 ± 51.9	587.3 ± 34.2

Values are mean and SD of three experiments. The differences between IL-6 concentrations in the *A. cruentus* treated medium and in the positive control medium were significant at *p* < 0.05

## Concluding Remarks

In the present paper, we have demonstrated that *A. cruentus* seeds and sprouts extracts treatment had anti-inflammatory effect on RAW 264.7 macrophages by preventing the TNF-α induced translocation of NFκB to the nucleus, which effect was followed by a decreased release of IL-6 from LPS activated cells. The biological effects of seeds and sprouts extracts were correlated with betacyanins concentrations in plants and sprouts ability to accumulate Se. None of the tested amaranth species decreases mammalian cells proliferation and viability. Though further studies are required to recognize the precise mechanisms, it can be concluded that Se supplemented amaranth sprouts may be considered as a novelty for potent functional food, which can contribute to the lower risk of chronic inflammation related diseases due to beneficial influence on NFκB signalling pathway. Amaranth seeds and sprouts may become a valuable source of betacyanin as an alternative to beetroot, and in addition to its unique biological and nutritive properties, attractively red-violet coloured amaranth sprouts may help to promote proper nutritional habits in consumers.

## Electronic Supplementary Material

ESM 1 Effect of amaranth seeds and sprouts extracts (A—*A. cruentus*; B—*A. caudatus*; C—*A. paniculatus*; D—*A. tricolor*) on RAW 264.7 cells viability (MTT assay performed after 24 h of incubation with a range of concentrations from 1 mg/mL to 0.1 μg/mL) (*n* = 3). (DOCX 70 kb)
