# Supplementary material for: Selenium Supplementation of Amaranth Sprouts Influences Betacyanin Content and Improves Anti-Inflammatory Properties via NFκB in Murine RAW 264.7 Macrophages
Source: Biol Trace Elem Res. 2015 Jul 12;169:320–30. doi: 10.1007/s12011-015-0429-x (PMC4717175; doi:10.1007/s12011-015-0429-x)
Supplement: Supplementary file 1 — Effect of amaranth seeds and sprouts extracts (A—A. cruentus; B—A. caudatus; C—A. paniculatus; D—A. tricolor) on RAW 264.7 cells viability (MTT assay performed after 24 h of incubation with a range of concentrations from 1 mg/mL to 0.1 μg/mL) (n = 3). (DOCX 70 kb) [file 12011_2015_429_MOESM1_ESM.docx]

Supplementary materials –Biological Trace Element Research

Malgorzata Tyszka-Czochara, Pawel Pasko, Pawel Zagrodzki, Ewelina Gajdzik, Renata Wietecha-Posluszny, Shela Gorinstein

Selenium supplementation of amaranth sprouts influences betacyanin content and improves anti-inflammatory properties via NFκB in murine RAW 264.7 macrophages

#corresponding author Pawel Pasko (PharmD) e-mail: paskopaw@poczta.fm; Department of Food Chemistry and Nutrition, Medical College, Jagiellonian University, 30-688 Krakow, Poland, Medyczna 9. Tel +48126205670; Fax +48126205693.


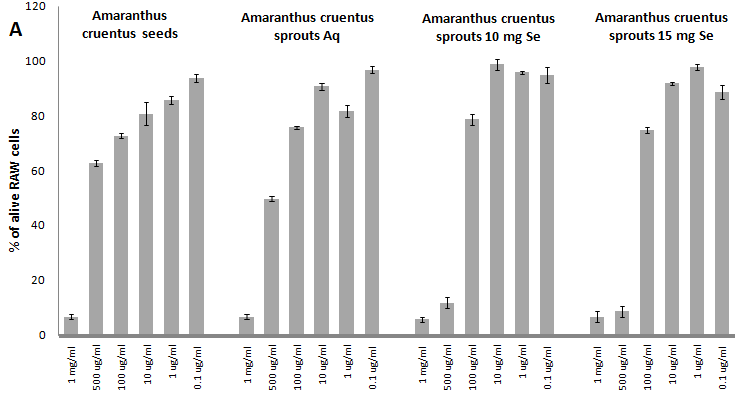


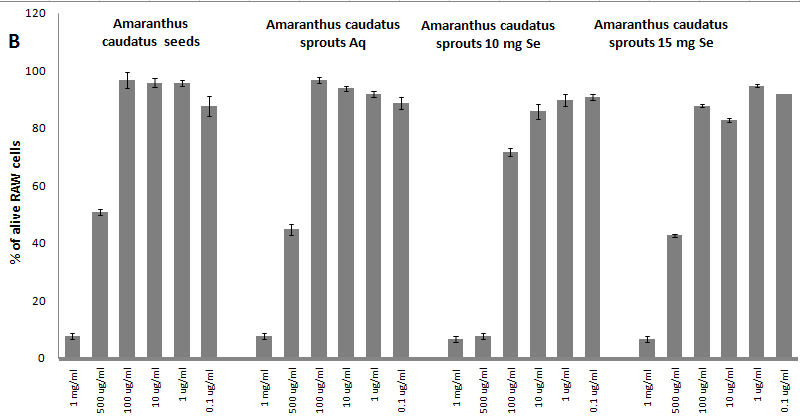


**
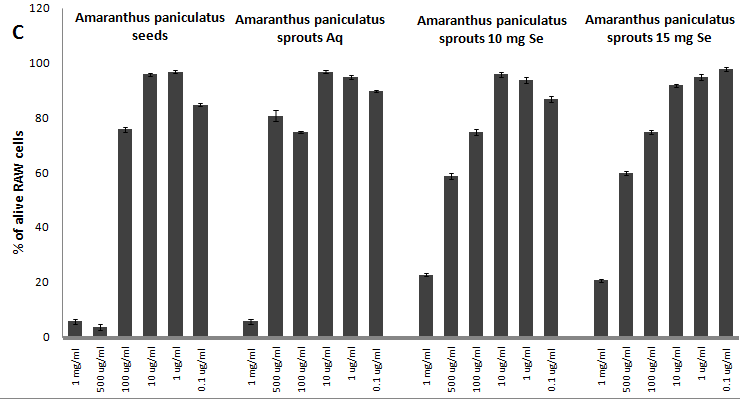
**

**
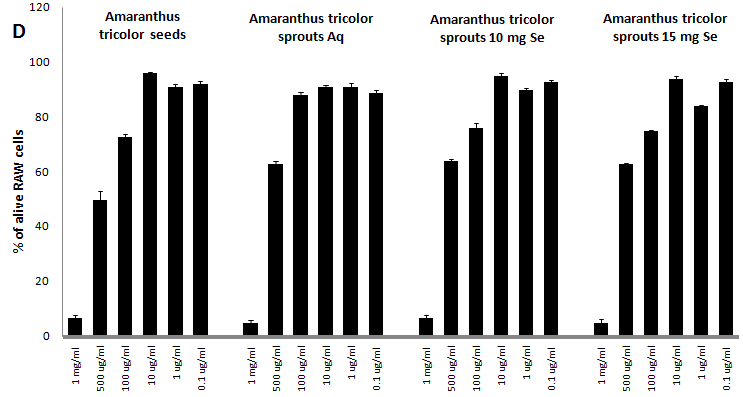
**

Effect of amaranth seeds and sprouts extracts (A - *A. cruentus*; B – *A. caudatus*; C- *A. paniculatus*; D – *A. tricolor*) on RAW 264.7 cells viability (MTT assay performed after 24 h of incubation with a range of extracts concentrations from 1 mg/mL to 0.1 μg/mL) (n=3).
